# A novel intervention combining supplementary food and infection control measures to improve birth outcomes in undernourished pregnant women in Sierra Leone: A randomized, controlled clinical effectiveness trial

**DOI:** 10.1371/journal.pmed.1003618

**Published:** 2021-09-28

**Authors:** David Taylor Hendrixson, Kristie Smith, Patrick Lasowski, Meghan Callaghan-Gillespie, Jacklyn Weber, Peggy Papathakis, Per Ole Iversen, Aminata Shamit Koroma, Mark J. Manary

**Affiliations:** 1 Department of Pediatrics, Washington University School of Medicine in St. Louis, Saint Louis, Missouri, United States of America; 2 Department of Food Science and Nutrition, California Polytechnic State University, San Luis Obispo, California, United States of America; 3 Department of Nutrition, University of Oslo, Oslo, Norway; 4 Ministry of Health and Sanitation, The Republic of Sierra Leone, Freetown, Sierra Leone; 5 Children’s Nutrition Research Center, Baylor College of Medicine, Houston, Texas, United States of America; The Hospital for Sick Children, CANADA

## Abstract

**Background:**

Innovations for undernourished pregnant women that improve newborn survival and anthropometry are needed to achieve the Sustainable Development Goals 1 and 3. This study tested the hypothesis that a combination of a nutritious supplementary food and several proven chemotherapeutic interventions to control common infections would increase newborn weight and length in undernourished pregnant women.

**Methods and findings:**

This was a prospective, randomized, controlled clinical effectiveness trial of a ready-to-use supplementary food (RUSF) plus anti-infective therapies compared to standard therapy in undernourished pregnant women in rural Sierra Leone. Women with a mid-upper arm circumference (MUAC) ≤23.0 cm presenting for antenatal care at one of 43 government health clinics in Western Rural Area and Pujehun districts were eligible for participation. Standard of care included a blended corn/soy flour and intermittent preventive treatment for malaria in pregnancy (IPTp). The intervention replaced the blended flour with RUSF and added azithromycin and testing and treatment for vaginal dysbiosis. Since the study involved different foods and testing procedures for the intervention and control groups, no one except the authors conducting the data analyses were blinded. The primary outcome was birth length. Secondary outcomes included maternal weight gain, birth weight, and neonatal survival. Follow-up continued until 6 months postpartum. Modified intention to treat analyses was undertaken. Participants were enrolled and followed up from February 2017 until February 2020.

Of the 1,489 women enrolled, 752 were allocated to the intervention and 737 to the standard of care. The median age of these women was 19.5 years, of which 42% were primigravid. Twenty-nine women receiving the intervention and 42 women receiving the standard of care were lost to follow-up before pregnancy outcomes were obtained. There were 687 singleton live births in the intervention group and 657 in the standard of care group. Newborns receiving the intervention were 0.3 cm longer (95% confidence interval (CI) 0.09 to 0.6; *p* = 0.007) and weighed 70 g more (95% CI 20 to 120; *p* = 0.005) than those receiving the standard of care. Those women receiving the intervention had greater weekly weight gain (mean difference 40 g; 95% CI 9.70 to 71.0, *p* = 0.010) than those receiving the standard of care. There were fewer neonatal deaths in the intervention (*n =* 13; 1.9%) than in the standard of care (*n* = 28; 4.3%) group (difference 2.4%; 95% CI 0.3 to 4.4), (HR 0.62 95% CI 0.41 to 0.94, *p* = 0.026). No differences in adverse events or symptoms between the groups was found, and no serious adverse events occurred. Key limitations of the study are lack of gestational age estimates and unblinded administration of the intervention.

**Conclusions:**

In this study, we observed that the addition of RUSF, azithromycin, more frequent IPTp, and testing/treatment for vaginal dysbiosis in undernourished pregnant women resulted in modest improvements in anthropometric status of mother and child at birth, and a reduction in neonatal death. Implementation of this combined intervention in rural, equatorial Africa may well be an important, practical measure to reduce infant mortality in this context.

**Trial registration:**

ClinicalTrials.gov NCT03079388.

## Abstract 2

In a randomized trial, D. Taylor Hendrixson and colleagues investigate the effect of an intervention combining supplementary food and anti-infection medications on birth outcomes.

## Author summary

**Why was this study done?:**

- Undernutrition in pregnancy increases the risk of adverse pregnancy outcomes.
- Interventions for undernutrition in pregnancy have been traditionally based on nutritional supplementation with modest improvement.
- Pregnant women with undernutrition are at increased risk of infection and inflammation.
- The effect of bundling nutritional and anti-infective interventions for undernutrition in pregnancy has not been implemented in a clinical trial.

**What did the researchers do and find?:**

- We performed a randomized, controlled clinical trial of a ready-to-use supplementary food (RUSF) plus anti-infective therapies compared to standard therapy for undernourished pregnant women in rural Sierra Leone.
- Infants born to women receiving the intervention had longer birth lengths, larger weights, and larger mid-upper arm circumferences (MUACs) when compared to infants born to mothers receiving the standard of care.
- Mortality was decreased among infants born to mothers receiving the intervention through 6 months of life.
- Women receiving the intervention had increased weight gain and greater recovery from undernutrition when compared to women receiving the standard of care.

**What do these findings mean?:**

- Bundling nutritional and anti-infective interventions for the treatment of undernourished pregnant women improved infant birth size, growth, and survival through 6 months of life.
- Futher studies are required to determine the effect of the intervention on preterm birth.

## Introduction

Undernutrition in pregnancy is associated with serious adverse risks for mothers and their unborn children. Maternal undernutrition occurs in 24% of pregnancies in sub-Saharan Africa [1]. Maternal undernutrition can be reliably identified by mid-upper arm circumference (MUAC). Maternal MUAC <23.0 cm is associated with increased risk of small-for-gestational age (SGA) infants [2–4]. The risk of childhood stunting and wasting is increased in SGA infants [5].

Effective management of maternal undernutrition could improve child survival, growth, and development [6–8]. Dietary supplements targeted to undernourished pregnant women have demonstrated modest clinical success [9–11]. Supplementary food increases maternal weight gain. Ready-to-use supplementary foods (RUSFs) may be superior because they also decrease the incidence of SGA and low birth weight in some contexts [11,12].

Reduction in exposures that provoke maternal inflammation also improve newborn growth. Common inflammatory stimuli in Sierra Leone and sub-Saharan Africa include malaria, sexually transmitted infections, and gut parasite infestation. *Plasmodium falciparum* infection is especially deleterious in pregnancy because of its tropism for the placenta, leading to stillbirth, SGA, and premature delivery [13,14]. Reduction in malaria exposure has been achieved by the use of insecticide-treated bednets and intermittent preventive treatment for malaria in pregnancy (IPTp) with sulfadoxine-pyrimethamine (SP) during the second and third trimesters [15]. Treatment of helminth infections is safe, reduces anemia, and potentially impacts congnitive and motor function in the offspring [16–19]. Interventions to reduce malaria and helminth exposure are recommended for all at-risk pregnant women by WHO.

Azithromycin is a broad-spectrum antibiotic with activity against *Chlamydia trachomatis*, *Neisseria gonorrhea*, *Haemophilus ducreyi*, and malaria. Azithromycin given during pregnancy reduced stunting, premature delivery, and postneonatal mortality; sustained improvements in growth and neurodevelopment were seen at 5 years of age in Malawi [20–22]. Bacterial vaginosis is a dysbiosis found in 38% to 51% of pregnant women in sub-Saharan Africa, and its presence is associated with preterm labor [23]. The dysbiosis resolves with the administration of metronidazole, and there is evidence to support testing and treatment of women in populations at high risk for preterm delivery [24,25].

This clinical trial, conducted in Sierra Leone, tested the hypothesis that a combination of an RUSF, enhanced IPTp, azithromycin administration, and testing and treatment for bacterial vaginosis when added to the standard of care in undernourished pregnant women improves newborn length and weight.

## Methods

### Study design

This was a prospective, randomized, controlled clinical effectiveness trial in undernourished pregnant women comparing the impact of a package of nutritional and anti-inflammatory interventions with the standard of care in Sierra Leone. The primary outcome was birth length in a liveborn singleton pregnancy.

A sample size of 1,514 undernourished pregnant women, divided equally between intervention and the standard of care, was estimated to allow for a 20% lost to follow-up and/or exclusion rate, leaving a final sample size of 1,200 (600 per group) with a two-tailed significance of 0.05, power of 80% to detect a difference of 0.22 SD or 0.5 cm in birth length and a difference of 0.19 SD or 80 g in birth weight. Full details of the study design and interventions administered have been described previously [26]. The study is reported as per the Consolidated Standards of Reporting Trials (CONSORT) guideline (S1 Fig).

### Participants

All pregnant women with undernutrition defined by a MUAC ≤23 cm and a fundal height <35 cm as a proxy for duration of gestation were invited to participate from 43 antenatal clinics in Pujehun and Western Rural Area Districts of Sierra Leone. Exclusion criteria were known gestational diabetes, hypertension, or severe anemia.

Informed consent was obtained for eligible and interested women, and this was documented by a signature or thumbprint if the participant was unable to write. Women older than 16 years of age were eligible to consent for themselves, and girls younger than the age of 16 desiring to participate required consent from a parent or guardian. Ethical approval was obtained from the Sierra Leone Ethics and Scientific Review Committee (SLESRC) and from the Human Research Protection Office at Washington University in St. Louis. The trial was registered at ClinicalTrials.gov (NCT03079388) on 14 March 2017, 16 days after the first participant was enrolled. Late trial registration was the result of very poor 2G internet access at the study site and a failure of those supporting the trial from abroad to perform this function while the first author was at the study site.

### Randomization and masking

Participants were randomized to intervention or standard of care using a numbered, computer-generated randomization list created for the entire study. A set of opaque envelopes containing each assignment was created from this list. The envelopes were placed in sets of 25, those numbered 1 to 25, 26 to 50, 51 to 75 and so forth constituted a set, and eligible women then selected an envelope from the set to determine their group assignment. No participant was allowed to choose a second envelope or exchange her envelope after initial selection. All envelopes in the set currently in use were chosen to assign a participant to a group before a second set was brought into use. Further details are available in S1 Text. The RUSF was visually distinct from flour, so neither the study participants nor the field research study team members were masked. Birth outcome assessor and study managers were masked to treatment during data collection and analysis.

### Participation

The study was conducted in conjunction with care at government-provided antenatal clinics. Upon enrollment, demographic information, time of last menses, and estimated date of delivery were recorded. A clinical symptom questionnaire was completed. Weight, height, MUAC, blood pressure, and fundal height were measured by trained study staff. Weight was measured with a Seca 803 digital scale (Hamburg, Germany). Height was measured with a Seca stadiometer (Hamburg, Germany). MUAC was measured on the left arm in centimeters to the nearest 10th of a centimeter with a flexible measuring tape (TALC), according to standard procedures. Fundal height was measured in the supine position with a nonelastic tape to the nearest 0.5 cm and used as a proxy for length of gestation [27]. Participants returned for follow-up every 2 weeks for anthropometric assessment and provision of the study foods and medications until delivery. Follow-up occurred at 6 weeks, at 3 months, and at 6 months after birth. Participants were considered lost to follow-up after missing 3 consecutive visits. Home visits were attempted for any patient lost to follow-up.

Clinic staff and participants were provided a telephone number and credit to call the study coordinator at the time of delivery. A birth measurement team was dispatched to measure infants within 48 hours of delivery. Birth measurements were taken as soon as was feasible, but because of poor road conditions and flooding during rainy season, this was not always possible. Infant survival, weight, length, head circumference, MUAC, morbidity, and feeding practices were assessed at each visit. Nude weight was obtained using Seca 334 infant digital scale (Hamburg, Germany) accurate to 5*g*. Recumbent length was measured in triplicate using a Seca 417 rigid height board (Hamburg, Germany); if the measurements differed by 2 mm, a fourth measurement was taken and the 3 closest measurements were recorded and averaged. Head circumference and MUAC of the left arm was obtained using a standard tape following standard procedures to the nearest 1 mm. Maternal weight, MUAC, and morbidity were assessed at these visits as well. If an infant was identified as deceased, an attempt was made to identify the cause of death by interviewing the mother and review of the medical record.

### Interventions

The RUSF development and production have been previously described [26]. Briefly, linear programming was used to optimize the RUSF for nutrient content, cost, and inclusion of indigenous ingredients. Four candidate formulations were tested for acceptability in antenatal clinics in Pujehun, Sierra Leone. The preferred formulation, named Mama Dutasi, contained skimmed milk powder, whey protein isolate, vegetable oil, sugar, peanut paste, and pearl millet. The daily RUSF ration provided 520 kcal, 18 g protein, and at least 100% of recommended daily allowance (RDA) for most micronutrients during pregnancy (S1–S3 Tables). The micronutrient premix included in the RUSF provided the same quantities of micronutrients as the UNICEF/WHO/United Nations multiple micronutrient supplement for pregnant and lactating women (UNIMMAP) with additional calcium and magnesium. The RUSF was produced by Project Peanut Butter, Sierra Leone. Each batch of food met UNICEF specifications for aflatoxin, *Enterobacter* sp., and *Salmonella* contamination. The standard of care group received 250 g/d of corn/soy blended flour (SuperCereal, World Food Programme) and 25 g palmolein oil daily. This provided 589 kcal, 17.5 g protein for the participant and as well as a ration for family sharing as is standard WFP practice. They also received 60 mg iron given as ferrous sulfate and 400 μg folic acid. Nutritional supplementation was initiated at the time of enrollment and continued until the time of delivery. Adherence with nutritional intervention was assessed at each visit using a standardized questionnaire.

The intervention group received 1,500 mg/75 mg of SP given every 4 weeks beginning in the second trimester. The standard of care group received 3 doses of 1,500 mg/75 mg SP during second and third trimesters.

The intervention group received 1 g of azithromycin at the beginning of the second trimester and in the third trimester. The intervention group was tested for vaginal dysbiosis upon enrollment and again in the third trimester using rapid sialidase testing (OSOM BVBLUE, Sekisui Diagnostics, Burlington, MA, USA). If dysbiosis was found, participants were treated with 500 mg metronidazole twice daily for 7 days.

All participants received an insecticide-treated bednet and 400 mg of albendazole in the second trimester of pregnancy. Doses of SP for IPTp, azithromycin, and albendazole were given under direct observation during study visits. Only the first dose of metronidazole was administered under direct observation. The study assured uninterrupted access to study food and medicine for the duration of their pregnancy.

### Outcomes

The primary outcome was newborn length. Secondary outcomes were the total and average weekly maternal weight gain, fundal height at delivery, recovery from maternal undernutrition defined as a MUAC >23 cm, newborn weight and head circumference, infant linear and ponderal growth through 6 months, and infant survival at 1.5, 3, and 6 months.

### Statistical analyses

Data were first recorded on clinic management cards. Data from these cards were then double entered into a spreadsheet database (Microsoft Access) and cross checked for discrepancies. All discrepancies were resolved by examination of the original data card. Once the content of the database was determined, it was locked for analyses.

Descriptive statistics were used to characterize the study population. Mothers with singleton live births were included in the modified intention-to-treat comparative analyses. All singleton live birth data were included in the primary comparison, regardless of maternal duration of participation or adherence. Those lost to follow-up prior to delivery could not be included. All anthropometric parameters were converted to z-scores using the 2006 WHO growth standards [28]. Two-sided independent samples Student *t* test was used to compare continuous outcomes (IBM SPSSS Statistics v. 25, IBM, Armonk, NY, USA). Mean differences with 95% confidence intervals (CIs) were calculated. A *p-*value of <0.05 was considered statistically significant for hypothesis testing. Fisher exact test was used to compare categorical outcomes, and Newcombe/Wilson score with continuity correction was used to calculate 95% CIs for differences in the proportions.

Linear mixed effects analysis of the relationship of the intervention and maternal and infant anthropometrics over the study period was performed in R (version 3.6.0) and *lme4* package. For maternal anthropometric analysis, fixed effects of study group, duration of enrollment, and their interaction were entered with individual participant variability accounted for as a random variable. For analysis of infant anthropometrics as fixed effects, we entered whether the mother/newborn dyad received the intervention or control, age at measurement, and sex, and their interaction with individual participant variability accounted for as a random variable. *p*-values were estimated via *t* tests using the Satterthwaite approximations to degrees of freedom.

Time–event analysis was performed to compare infant survival up to 6 months of age between the groups. The Cochran–Mantel–Haenszel test was used to calculate hazard ratios (HRs). Statistical analysis was performed using IBM SPSS Statistics v. 25 (IBM, Armonk, NY, USA), R (version 3.6.0), and GraphPad Prism version 8.3.0 (GraphPad, San Diego, CA, USA).

## Results

**Fig 1:**
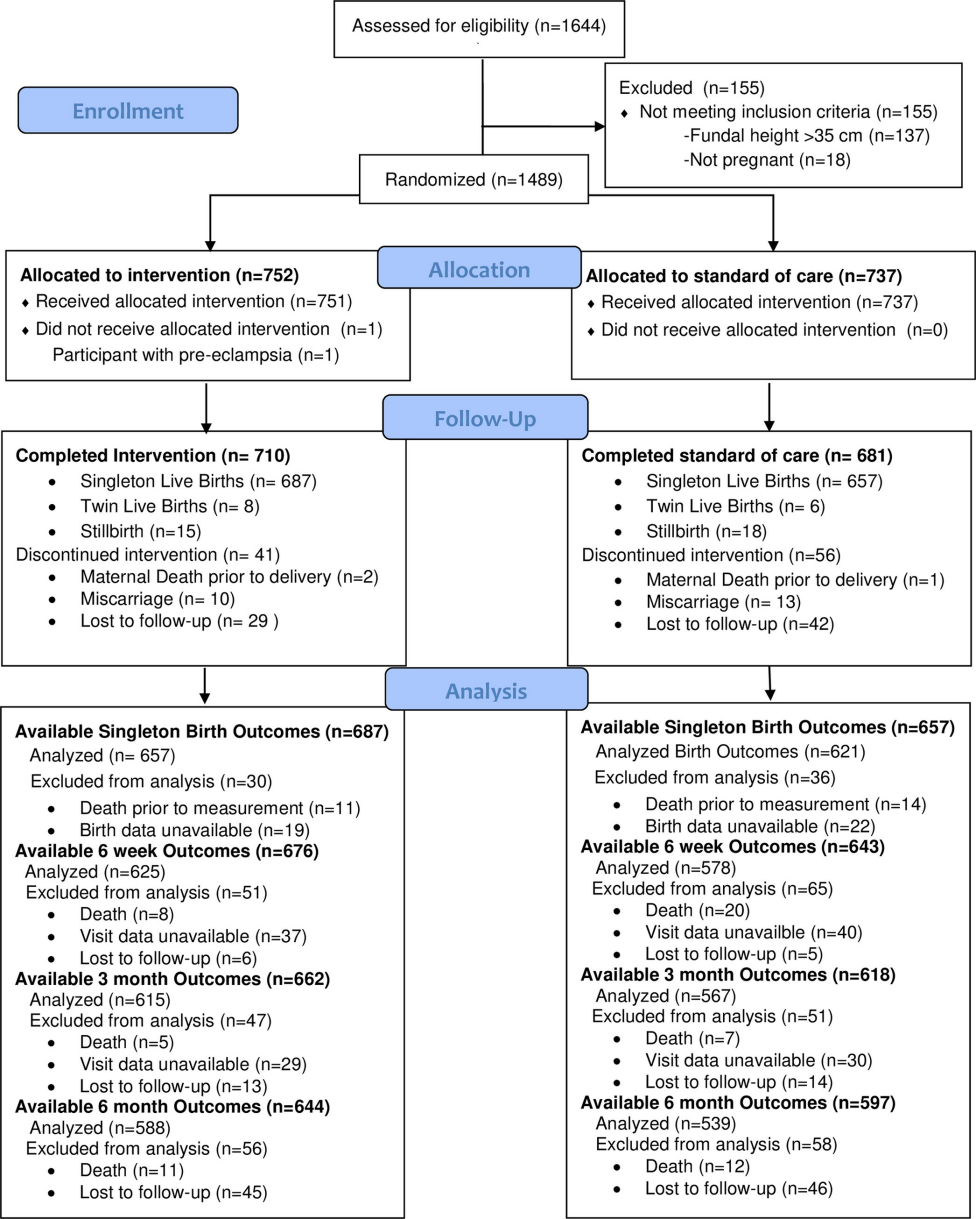
CONSORT flow diagram. “Lost to follow-up” refers to individuals who were not seen at the time point indicated and subsequently never seen again. “Visit data unavailable” refers to individuals who were not seen at the time point indicated but subsequently seen. Deaths are reported as additional deaths between each time point.

Of the 1,489 women enrolled in the study from February 2017 to May 2019, 752 were allocated to the intervention group and 737 to the standard of care group (Fig 1). The numbers of women that defaulted were low with 29 (3.9%) receiving the intervention and 42 (5.7%) receiving the standard of care (Table 1). The duration of participation was 15.4 weeks (Q1:10, Q3:21) for the intervention and 14.9 weeks (Q1:9.3, Q3:20) for the standard of care (median difference 0.5; 95% CI −0.29 to 1.43).

**Table 1:** Enrollment characteristics^1^.

Characteristic	Intervention *n* = 751	Standard *n* = 737
Age^2^, year	21.0 ± 4.7	21.5 ± 4.9
Age^2^, median (range)	19.0 (13–40)	20.0 (14–42)
Participant <18 years of age	294 (40)	244 (34)
First pregnancy	335 (45)	290 (39)
Education		
None Primary Junior secondary Senior secondary Tertiary	234 (31) 153 (20) 218 (29) 138 (18) 8 (1)	247 (34) 161 (22) 200 (27) 123 (17) 6 (1)
MUAC, cm	22.3 ± 0.7	22.3 ± 0.8
Weight^3^, kg	48.1 ± 5.0	47.8 ± 4.9
Fundal height^4^, cm	23 ± 7	24 ± 6
Baseline height^5^, cm	155.8 ± 7.2	155.6 ± 6.4
Height <145 cm	30 (4)	29 (4)
HIV-infected^6^	25 (5)	26 (5)

MUAC, mid-upper arm circumference.

^1^Values expressed as mean ± SD or *n* (%).

^2^Age was unknown for Intervention *n =* 21 and Standard *n* = 24 participants.

^3^Initial weight missing in Intervention *n* = 2.

^4^Fundal height unmeasurable in Intervention *n* = 64, Standard *n* = 54.

^5^Baseline height missing in Intervention *n* = 4 and Standard *n* = 1.

^6^HIV status unknown in Intervention *n* = 235 and Standard *n* = 219.

There were no serious adverse events during the study period and no statistically significant differences in adverse events/symptoms between the groups (S4 Table). Adherence with the study foods was high in both groups as assessed by surveys (S5 Table). Adherence with IPTp was high with women in the intervention group receiving a mean of 4.4 doses (SD 1.4) and women in the standard group receiving 2.8 doses (SD 0.6) (S6 Table). Azithromycin adherence was good among women in the intervention group with 473 (68.9) receiving 2 doses and 210 (30.6%) receiving 1 dose prior to delivery. The timing of last azithromycin administration was on average 8.2 (SD 3.8) weeks before delivery. There were no differences in the number of maternal deaths, miscarriages, or stillbirths (S7 Table). There were 687 (91.5%) singleton live births in the intervention group and 657 (89.2%) in the standard of care group (*p* = 0.136).

When compared to women receiving the standard of care, women receiving the intervention had greater total weight gain, weekly weight gain, larger MUAC gain from enrollment, and had a 7.2% higher rate of recovery from undernutrition at final study visit (Table 2). The number needed to treat for one woman to recover from undernutrition was 13.9 (95% CI 8.3 to 44.2) Fewer women in the intervention group than the standard of care group delivered with fundal height <37 cm (difference 10.5%; 95% CI 06.3 to 14.6, *P* ≤ 0.001) (Table 2, S1 Fig).

**Table 2:** Maternal outcomes for mothers with singleton live births, by treatment group^1^.

Outcome	Intervention *n* = 687	Standard of Care *n* = 657	*P*	Mean Difference (95% CI)
Weeks enrolled^2^	15.4 (Q1:10, Q3:21)	14.9 (Q1:9.3, Q3:20)	0.183	0.5(−0.29 to 1.43)
MUAC at final antenatal visit, cm	22.8 ± 1.0	22.6 ± 1.2	**0.005**	0.17(0.05 to 0.28)
Change in MUAC, cm	0.5 ± 0.8	0.4 ± 0.9	**0.015**	0.11(0.02 to 0.20)
Weight at final antenatal visit, kg	53.68 ± 5.11	52.56 ± 5.08	**<0.001**	1.12(0.57 to 1.66)
Total weight gain, kg^3^	5.36 ± 3.75	4.69 ± 3.67	**0.001**	0.67(0.27 to 1.06)
Average weekly weight gain, g^3^	379 ± 222	339 ± 333	**0.010**	40(10 to 71)
Weight gain <454 g/week^3^	457(68.7)	477(75.0)	**0.014**	6.3%(1.3 to 11.3)
Final fundal height^4^, cm	36.1 ± 2.6	35.6 ± 2.9	**0.007**	0.4(0.1 to 0.7)
Final fundal height <37 cm^5^	528(77.3)	574(87.7)	**<0.001**	10.5%(6.3 to 14.6)
Final fundal height <28 cm^4^	11(1.6)	16(2.4)	0.277	0.8%(−0.9 to 2.5)
Final fundal height 28 to 32 cm^4^	62(9.1)	85(13.0)	**0.022**	3.9%(0.4 to 7.4)
Final fundal height 32 to 37 cm^4^	455(66.6)	473(72.3)	**0.024**	5.7%(0.7 to 10.8)
MUAC at delivery^6^, cm	22.5 ± 1.0	22.3 ± 1.2	**<0.001**	0.2(0.1 to 0.4)
Recovered from undernutrition^6^	198(29.9)	142(22.7)	**0.004**	7.2%(2.3 to 12.0)

MUAC, mid-upper arm circumference.

^1^Values expressed as mean ± SD or *n* (%); *p-*values calculated using independent *t* test (continuous measures), Fisher exact test, or chi-squared test (categorical measures). For outcomes reported as numbers and percentages of participants, the difference is given as the percentage–point difference between groups.

^2^Values expressed as median (quartiles); *p-*value calculated using Mann–Whitney *U* test.

^3^Initial weight unavailable in Intervention *n =* 1. Only 1 study visit Intervention *n* = 21, Standard *n* = 21.

^4^Final fundal height not available for Intervention *n* = 4 and Standard *n* = 3.

^5^Fundal height was used as a crude marker for gestational age. Birth <28 weeks is considered extremely preterm, 28 to 32 weeks is very preterm, and 32 to 37 weeks is late preterm.

^6^MUAC a delivery not available for Intervention *n* = 24 and Standard *n* = 31.

Using linear mixed modeling, the intervention was shown to increase weight gain from enrollment when the duration of participation was included as a covariate resulting in an increase of 30 grams (SE 10) of weight gain per week of enrollment (*p* = <0.001; 95% CI 20 to 40) (S8 Table). MUAC change was increased in women in the intervention group when compared to standard of care. Over the course of enrollment, women in the intervention group gain 0.1 cm (SE 0.04) more in MUAC (*p* = <0.001; 95% CI 0.1 to 0.2) or 0.01 cm (SE 0.002) per week enrolled (*p* = <0.001; 95% CI 0.01 to 0.01) (S8 Table).

When evaluating postpartum maternal outcomes, linear mixed modeling demonstrated a significant effect of the study group and time since birth on maternal weight with mothers in the intervention group weighing 0.81 kg (SE 0.28) more than mothers in the standard of care group (*p* = 0.003; 95% CI 0.27 to 1.36) (S9 Table). The effect of the intervention appeared to wane with time since birth. Maternal postpartum MUAC was not significantly different between women in the 2 groups (S9 Table).

Among singleton live births, there were 657 infants in the intervention and 621 in the standard of care groups with available birth data. Birth measurements were obtained at median of 1.0 days (Q1:1.0 and Q3:3.0) both the intervention group and the standard of care group (median difference 0.0; 95% CI 0 to 0; *p* = 0.968). Infants born to mothers in the intervention group were 0.3 cm longer (95% CI 0.09 to 0.6; *p* = 0.007), weighed 70 g more (95% CI 20 to 120; *p* = 0.005), and had MUACs that were 0.1 cm larger (95% CI 0.03 to 0.2; *p* = 0.006) than infants born to mothers receiving the standard of care (Table 3).The effect size remained the same for birth length when only infants measured within 7 days were considered (0.3 cm; 95% CI 0.002 to 0.5; *p* = 0.049) (S10 Table). Among the 41 participants with missing birth data, 19 (46%) were in the intervention group and 22 (54%) were in the standard of care group. Of the 113 infants with birth data collected after 7 days of life, 62 (55%) were in the intervention group and 51 (45%) were in the standard of care group.

**Table 3:** Singleton infant outcomes, by treatment group^1^.

	Intervention	Standard		
Outcome	Value	Value	*P*	Mean Difference (95% CI)
Sex	*n* = 676	*n* = 643	0.322	
Female Male	332(49.2) 343(50.8)	334(51.9) 309(48.1)		2.8(−2.7 to 8.3)
	*n* = 657	*n* = 621		
Birth length, cm	47.2 ± 2.2	46.9 ± 2.5	**0.007**	0.3(0.1 to 0.6)
Birth weight, kg	2.87 ± 0.44	2.80 ± 0.44	**0.005**	0.07(0.02 to 0.12)
Birth head circumference, cm	33.9 ± 1.4	33.8 ± 1.6	0.109	0.1(−0.03 to 0.3)
Birth MUAC, cm	9.9 ± 0.8	9.8 ± 0.8	**0.006**	0.1(0.03 to 0.2)
Birth LFA, z-score^1^	−1.45 ± 1.06	−1.57 ± 1.16	0.054	0.12(−0.002 to 0.24)
Birth WFL, z-score^2^	0.03 ± 1.1	−0.01 ± 1.02	0.478	0.05(−0.08 to 0.18)
Birth WFA, z-score	−1.01 ± 0.96	−1.15 ± 1.03	**0.012**	0.14(0.03 to 0.25)
Birth BMI, z-score	−0.48 ± 1.11	−0.57 ± 1.03	0.152	0.09(−0.03 to 0.20)
Stunted at birth^1^, LFA < −2	184(31.1)	188(33.4)	0.414	2.3(−3.2 to 7.8)
Undernourished at birth^2^, WFL < −2	18(3.0)	16(2.8)	1.00	0.2(−2.5 to 2.7)
Underweight at birth, WFA < −2	80(13.5)	84(14.8)	0.556	1.3(−2.3 to 5.4)
Low birth weight (<2.5 kg)	115(19.5)	126(22.2)	0.248	2.7(−2.0 to 7.6)

BMI, body mass index; LFA, length-for-age z-score; MUAC, mid-upper arm circumference; WFA, weight-for-age z-score; WFL, weight-for-length z-score.

Values expressed as mean ± SD or *n* (%); *p-*values calculated using independent *t* test (continuous measures), Fisher exact test, or chi-squared test (categorical measures). For outcomes reported as numbers and percentages of participants, the difference is given as the percentage–point difference between groups.

^1^Infants had length ≤38.0 cm, too short for LFA z-score, Standard *n =* 4.

^2^Infants were excluded from WFL z-score because they were too short (<45.00 cm), Intervention *n* = 90 and Standard *n* = 112.

Linear mixed models demonstrated an effect of age at measurement, sex, and study group on the length, weight, and MUAC of infants born to mothers in the study (S11 Table). Infants in the intervention group were 0.3 cm (SE 0.1) longer than those in the standard of care group (*p* = 0.011; 95% CI 0.1 to 0.5) and 85*g* (SE 33) heavier than those in the standard of care group (*p* = 0.01; 95% CI 20 to 150) (S11 Table). Infant head circumference was not different between infants born to mothers in the 2 study groups.

**Fig 2:**
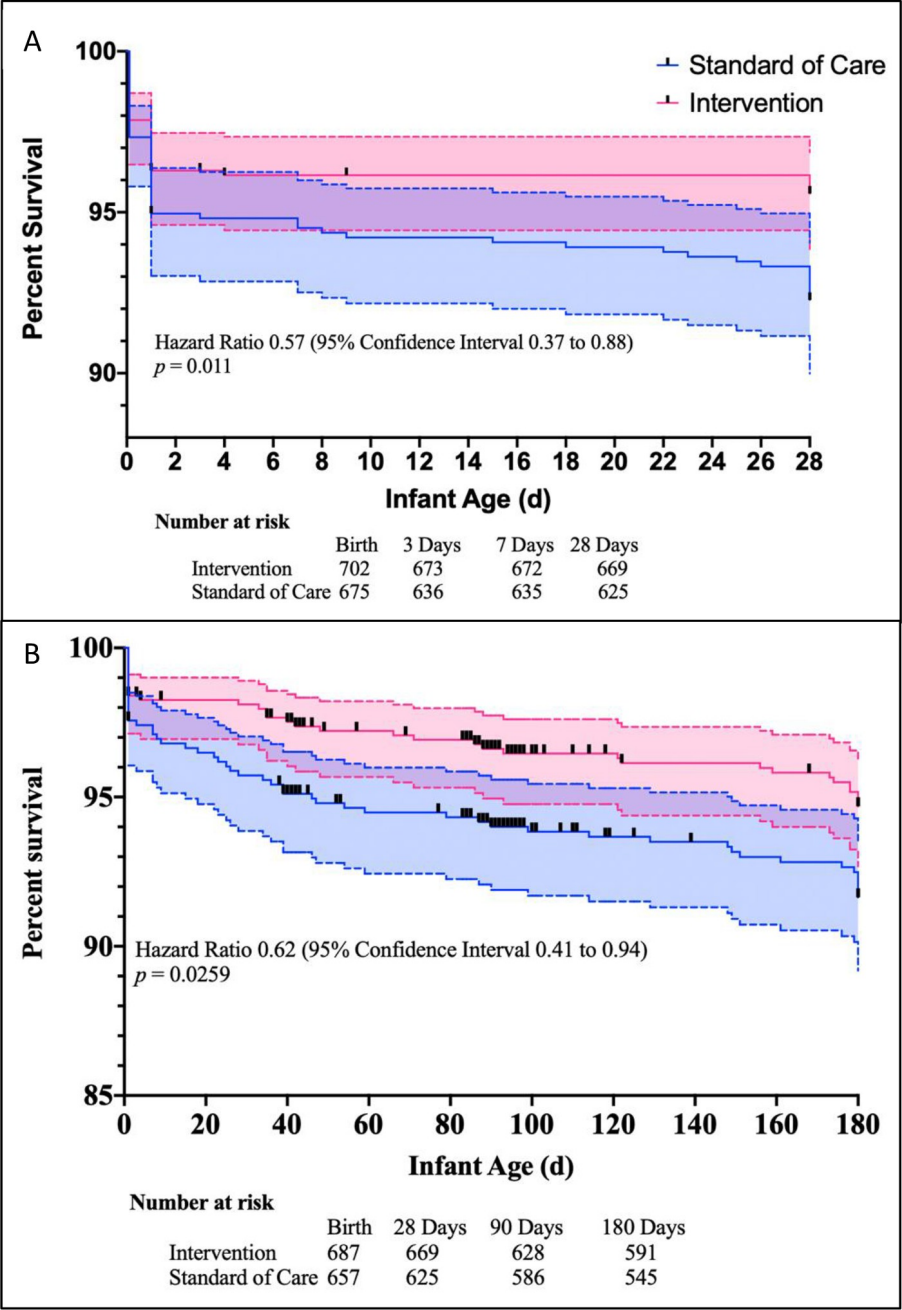
Kaplan–Meier survival estimates. Shown is the incidence of death among offspring assigned to the intervention group and those assigned to the standard of care. Values were calculated with the use of Kaplan–Meier methods and compared with Mantel–Haenszel test. Panel A demonstrates the survival curve for perinatal and neonatal mortality. Panel B demonstrates infant mortality from birth until 6 months of life.

There were fewer infant deaths in the intervention group (35; 5.6%) than in the standard of care group (53; 8.9%; difference 3.3%, 95% CI 0.3% to 6.4%, *p* = 0.027) in the first 6 months of life (S12 Table). The number needed to treat for infant survival benefit was 29.99 (95% CI 15.68 to 463.2). The HR for death among infants born to mothers in the intervention group was 0.62 (95% CI 0.41 to 0.94, *p* = 0.026) (Fig 2). The mortality benefit occurred during the neonatal period (≤21 days) when there were 13 (1.9%) deaths among the intervention group infants and 28 (4.3%) deaths among the standard of care group infants (difference 2.4%, 95% CI 0.3 to 4.4, *p* = 0.016; HR 0.44, 95% CI 0.23 to 0.82, *p* = 0.011) (S12 Table).

Among women in the intervention group, 370 (59%) tested positive for bacterial vaginosis. Among the 293 who had repeat testing prior to delivery, 217 (74%) remained positive for bacterial vaginosis despite treatment. There were no significant differences in pregnancy or growth outcomes between women testing positive and receiving treatment and those testing negative, except for increased maternal weight and MUAC gain in those testing negative and a slightly larger infant head circumference (S13 and S14 Tables).

The intervention cost an additional $13.86 per woman plus $0.21 per day for the RUSF when compared to the standard of care. This cost included testing for vaginal dysbiosis, which was $11.07.

## Discussion

In this study, we observed that infants born to undernourished women receiving the package of RUSF with a combination of anti-infective interventions were slightly longer and heavier, as well as more likely to survive the neonatal period when compared to those receiving the standard of care. Women receiving the intervention had greater weight gain when compared to those receiving the standard of care.

Other trials of combined nutritional and anti-infective interventions in undernourished pregnant women have not been reported to our knowledge.

The RUSF included milk as an ingredient; increased milk intake has been associated with increased birth weight [29–31]. RUSF is a balanced protein energy supplement, providing ample amounts of micronutrients and substantial quantities of essential amino acids required in pregnancy that were not provided in the blended flour provided to the standard of care group. Balanced protein energy supplementation has demonstrated modest increases in birth weight when provided to undernourished pregnant women [9,32]. A similar effect on birth length has been observed in some, but not all, trials distributing nutritional supplementation to undernourished pregnant women in Africa [9,33].

Neonatal deaths were reduced by the combined intervention. The major causes of neonatal deaths in this setting are prematurity, complications of delivery, and infant infection [34]. Azithromycin targets the ribosomal subunit of susceptible microbes, inhibiting protein synthesis and causing microbial death. Ureaplasma infections of the placenta are common, asymptomatic, associated with preterm birth and SGA newborns, and are effectively treated with azithromycin [35]. In Sierra Leone, malaria parasitemia in children under 5 years is about 47%, without significant seasonal variation [36]. Azithromycin has anti-inflammatory properties that may work to control maternal inflammation and improve fetal growth [37–40]. Azithromycin administration also results in alterations in the gut microbiome and perhaps may remove pro-inflammatory flora from the microbiota as a mechanism of action [41]. The most plausible mechanism of action for azithromycin in our study was the reduction of microbes harbored in the placenta, in particular malaria.

Four large clinical trials have reported the effects of using azithromycin in pregnancy given many weeks prior to delivery (S15 Table). Two of these were in Malawi, one that showed no effect on premature delivery, newborn anthropometry, maternal malaria parasitemia, or perinatal survival [42]. The other Malawian trial demonstrated a reduction in premature delivery, maternal parasitemia, and low birth weight, with a reduction in infant mortality after the neonatal period [43]. A third trial from Uganda found reduction in sexually transmitted diseases, low birth weight, and neonatal deaths within 7 days of delivery [44]. The fourth trial from Papua New Guinea found a reduction in premature delivery, low birth weight, and multiple measures of malaria infection [45]. The rates of neonatal death were very low in the control group in the trial from New Guinea. Mass azithromycin distribution reduced child mortality when given to children in Niger, Tanzania, and Malawi, with the largest effect in Niger where mortality is the highest [46,47]. Our data add to the multiple lines of evidence suggesting azithromycin may be an important adjunct to improve child survival.

Concern about the risk for emergence of resistant bacteria with widespread administration of azithromycin is frequently raised. Though use of azithromycin in pregnancy and labor increases the number of macrolide-resistant organisms identified in young infants, this resistance wanes after 12 months [48–50]. In undernourished pregnant women, the emergence of antibiotic resistant strains must be balanced against the opportunity to reduce neonatal death by 2.3-fold. In this study, azithromycin was first given in the second trimester, as recent epidemiologic evidence found an increased risk of major malformations in infants born to mothers prescribed a macrolide in the first trimester [51].

Vaginal dysbiosis did not resolve with metronidazole treatment in most cases. Recurrence of vaginal dysbiosis after treatment is common and is increased by continued exposure through sexual activity [52–54]. In other settings, more success in resolution of the dysbiosis has been seen with transplantation of vaginal secretions [55]. Chronic suppressive therapy has been demonstrated effective to reduce development or recurrence of bacterial vaginosis, though this approach cannot be applied broadly to a population without further investigation and evidence [56].

Vaginal dysbiosis testing and treatment with metronidazole as a component of large-scale intervention is not supported by our data.

The randomized design that enrolled a common cadre of high-risk pregnant women from a malaria-endemic, resource-limited setting is the greatest strength of the study. The intervention package was feasible; its elements have been used extensively in clinical settings. The implementation of the study might serve as an example for future operational programs. All outcomes were determined using standardized, objective methods, and birth outcomes were masked. The major limitation of this study was that there was no ultrasound dating of gestational age. Thus, we cannot determine whether the intervention reduced premature delivery. The unblinded design of the study introduced the possibility that participants shared their assigned food; mitigating this risk was that most participants resided more than 2 km apart. Participants receiving the standard of care may well have had improved outcomes when compared to women receiving typical clinical care, because of sporadic availability of any foods and medications in the resource-limited setting. The causes of infant death were not ascertained using reliable methods; therefore, conclusions cannot be drawn. The study population is representative of rural mothers in agrarian, malaria-endemic sub-Saharan Africa, and the effectiveness of the intervention cannot be reasonably extrapolated to other populations. This intervention was not given as a blanket distribution for all pregnant women living in a vulnerable environment, but rather targeted to those who were undernourished. Thus, no evidence for the use of the intervention as a universal public health practice can be inferred.

The intervention cost more when compared to the standard of care, which must be considered when considering scale-up of the intervention. The opportunity cost to participants returning to clinic fortnightly was high, but, operationally, food and medication administration could be done monthly.

Further studies are required to elucidate the effects of routine prenatal azithromycin on maternal and infant carriage of resistant organisms.

In conclusion, delivery of a bundled intervention of a nutritious RUSF and enhanced prevention of infection during pregnancy resulted in larger infants, improved maternal recovery from undernutrition, and improved neonatal survival. The intervention is promising as it could contribute to Sustainable Development Goal 3.2, reducing neonatal mortality to 25 per 1,000 live births in areas where neonatal death is high as well as risk for malaria infection [57].

## Supporting information

S1 Text Randomization, data monitoring, and data management details. (DOCX)

S1 Fig CONSORT Checklist with identified locations of required information. (DOCX)

S1 Table Ready-to-use supplementary food ingredients. (DOCX)

S2 Table Nutrient composition of study foods. (DOCX)

S3 Table Essential amino acids provided in the dietary supplements. (DOCX)

S4 Table Maternal symptoms over the first 4 weeks of enrollment, by treatment group. (DOCX)

S5 Table Adherence with study foods by study visit. (DOCX)

S6 Table Adherence with azithromycin and IPTp among pregnancies with singleton live births. (DOCX)

S7 Table Pregnancy outcomes by treatment group. (DOCX)

S8 Table Linear mixed model results of maternal antenatal anthropometric outcomes. (DOCX)

S9 Table Linear mixed model results of maternal postpartum anthropometric outcomes. (DOCX)

S10 Table Infant anthropometric outcomes by chronologic age. (DOCX)

S11 Table Linear mixed modeling of infant secondary outcomes to 6 months of age. (DOCX)

S12 Table Infant mortality among singleton infants by treatment group. (DOCX)

S13 Table Maternal and pregnancy outcomes by vaginal dysbiosis status among the intervention group. (DOCX)

S14 Table Infant birth outcomes by maternal vaginal dysbiosis status among the intervention group. (DOCX)

S15 Table Trials giving azithromycin in pregnancy with >1,200 participants in low- and middle-income settings. (DOCX)

## Abbreviations

CI: confidence interval
CONSORT: Consolidated Standards of Reporting Trials
HR: hazard ratio
IPTp: intermittent preventive treatment for malaria in pregnancy
MUAC: mid-upper arm circumference
RDA: recommended daily allowance
RUSF: ready-to-use supplementary food
SGA: small-for-gestational age
SLESRC: Sierra Leone Ethics and Scientific Review Committee
SP: sulfadoxine-pyrimethamine
UNIMMAP: UNICEF/WHO/United Nations multiple micronutrient supplement for pregnant and lactating women
