# Supplementary material for: A novel intervention combining supplementary food and infection control measures to improve birth outcomes in undernourished pregnant women in Sierra Leone: A randomized, controlled clinical effectiveness trial
Source: PLoS Med. 2021 Sep 28;18(9):e1003618. doi: 10.1371/journal.pmed.1003618 (PMC8478228; doi:10.1371/journal.pmed.1003618)
Supplement: S1 Text — (DOCX) [file pmed.1003618.s001.docx]

**S1 Text: Randomization, data monitoring and data management**

**RANDOMIZATION PROCEDURE**

An ordered list of 1600 assignments was computer generated by a spreadsheet which randomly selected one of 2 colors. A set of opaque envelopes containing a singled colored card was created from this list. The envelopes were placed in groups of 25, eligible women then selected an envelope to determine their group assignment. The color of the card could not be perceived through the sealed envelope and each group of 25 envelopes was completely selected before a new group of 25 envelopes was brought into use. One of the colors was designated for the intervention group and one for the control group by a study member not engaged in delivering clinical care. The RUSF was visually distinct from standard of care corn flour, so neither the study subjects nor the field research study team members were blinded. Study managers were blinded to treatment during data analysis.

**DATA MONITORING POLICY**

The primary investigator was responsible for the overall management of the trial. The on-site investigator was responsible for day to day management of clinic sites. Participants were instructed to report all rashes, emesis, diarrhea, or possible food allergy to local health workers. A data safety monitoring meeting was held every other month to review routine morbidity data, enrollment data, and participant satisfaction.

**DATA MANAGEMENT**

Clinical data were collected by research team members using standardized forms. Completed data forms were stored in a secure locked central location. All data was double entered, compared, resolved for discrepancies and sealed in a password-protected electronic database. The data set was locked before the randomization code was broken.
