# Supplementary figures and images for: A novel intervention combining supplementary food and infection control measures to improve birth outcomes in undernourished pregnant women in Sierra Leone: A randomized, controlled clinical effectiveness trial
Source: PLoS Med. 2021 Sep 28;18(9):e1003618. doi: 10.1371/journal.pmed.1003618 (PMC8478228; doi:10.1371/journal.pmed.1003618)

**
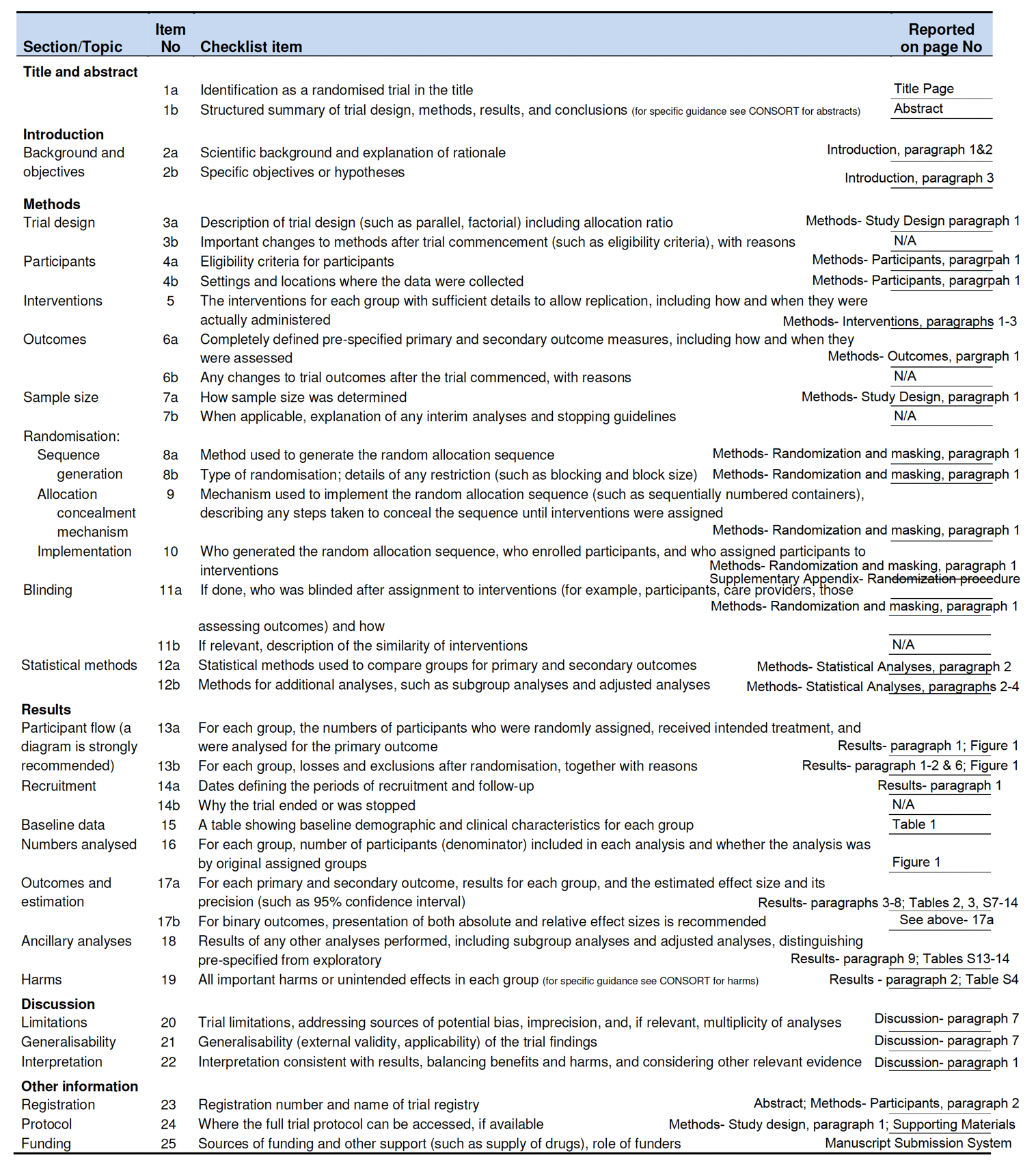
**

**S1 Figure**. CONSORT Checklist with locations of required information.

Supplement: S1 Fig — (DOCX) [file pmed.1003618.s002.docx]
