# Supplementary material for: A novel intervention combining supplementary food and infection control measures to improve birth outcomes in undernourished pregnant women in Sierra Leone: A randomized, controlled clinical effectiveness trial
Source: PLoS Med. 2021 Sep 28;18(9):e1003618. doi: 10.1371/journal.pmed.1003618 (PMC8478228; doi:10.1371/journal.pmed.1003618)
Supplement: S1 Table — (DOCX) [file pmed.1003618.s003.docx]

**S1 Table.** Ready-to-use supplementary food ingredients

| **Ingredient** | **Ready-to-use supplementary food** |
| --- | --- |
| Pearl millet, g/100g | 7.5 |
| Non-fat dry milk, g/100g | 21.5 |
| Whey protein isolate, g/100g | 6.8 |
| Palm oil, g/100g | 2.2 |
| High oleic soybean oil, g/100g | 25.6 |
| Brown sugar, g/100g | 20 |
| Peanut, g/100g | 10 |
| Multiple micronutrient premix, g/100g | 4.37 |
| Hydrogenated soy oil, g/100g | 2.00 |
