# Supplementary material for: A novel intervention combining supplementary food and infection control measures to improve birth outcomes in undernourished pregnant women in Sierra Leone: A randomized, controlled clinical effectiveness trial
Source: PLoS Med. 2021 Sep 28;18(9):e1003618. doi: 10.1371/journal.pmed.1003618 (PMC8478228; doi:10.1371/journal.pmed.1003618)
Supplement: S2 Table — (DOCX) [file pmed.1003618.s004.docx]

**S2 Table.** Nutrient composition of study foods

| **Ingredient** | **Ready-to-use supplementary food 100g** | **Corn/ soy blended flour with oil, iron/folic acid^a,b^** | **Recommended daily allowance^1-3^** |
| --- | --- | --- | --- |
| **Nutrient (units)** |  |  |  |
| Energy (Kcal) | 520 | 589 |  |
| Protein (g) | 18 | 17.5 |  |
| Fat (g) | 33 | 22.5 |  |
| n6 Polyunsaturated fatty acids (g) | 3.1 | 1.2 |  |
| n3 Polyunsaturated fatty acids (g) | 0.65 | 0 |  |
| Vitamin A (μg) | 770 | 1311 | 770 |
| Vitamin B1/Thiamine (mg) | 2.8 | 0.25 | 1.4 |
| Vitamin B2/Riboflavin (mg) | 2.8 | 1.75 | 1.4 |
| Vitamin B3/Niacin (mg) | 32 | 10 | 18 |
| Vitamin B6 (mg) | 3.8 | 1.25 | 1.9 |
| Vitamin B12 (μg) | 5.2 | 2.5 | 2.6 |
| Folic acid (μg) | 500 | 137.5 | 400 |
| Vitamin C (mg) | 170 | 112.5 | 85 |
| Vitamin D (μg) | 30 | 13.8 | 15 |
| Vitamin E (mg) | 30 | 10.4 | 15 |
| Iron (mg) | 30 | 33.25 | 27 |
| Zinc (mg) | 22 | 6.25 | 11 |
| Calcium (mg) | 1600 | 452.5 | 1000 |
| Chromium (μg) | 60 | - | 30 |
| Copper (μg) | 2000 | - | 1000 |
| Iodine (μg) | 300 | 50 | 220 |
| Magnesium (mg) | 300 | - | 350 |
| Phosphorus (mg) | 687.5 | 290 | 700 |
| Potassium (mg) | 892.7 | 140 | 4700 |
| Selenium (μg) | 120 | - | 60 |

Abbreviations: WFP, World Food Program

^a^ Daily ration: 250 gm supercereal (CSB+) with sugar, 20 ml oil, 60 mg Fe supplement with 400 mcg folic acid. WFP ration includes provision for sharing with anticipated consumption 50% of provided ration.

^b^ Calculated based on a WFP supercereal product containing ~64% corn , ~24% soybean,~25g vegetable oil

References:

1. Otten J, Hellwig J, Meyers L, editors. *Dietary reference intakes: the essential guide to nutrient requirements*. Washington, DC, USA: National Academy of Sciences, 2006.

2. . Dietary Reference Intakes for Vitamin A, Vitamin K, Arsenic, Boron, Chromium, Copper, Iodine, Iron, Manganese, Molybdenum, Nickel, Silicon, Vanadium, and Zinc. Washington (DC)2001.

3. . Dietary Reference Intakes for Vitamin C, Vitamin E, Selenium, and Carotenoids. Washington (DC)2000.
