# Supplementary material for: A novel intervention combining supplementary food and infection control measures to improve birth outcomes in undernourished pregnant women in Sierra Leone: A randomized, controlled clinical effectiveness trial
Source: PLoS Med. 2021 Sep 28;18(9):e1003618. doi: 10.1371/journal.pmed.1003618 (PMC8478228; doi:10.1371/journal.pmed.1003618)
Supplement: S3 Table — (DOCX) [file pmed.1003618.s005.docx]

**S3 Table**. Essential amino acids provided in the dietary supplements^1^

| **Amino acid** | **Ready-to-use supplementary food 100g** | **Corn/ soy blended flour with oil, iron/folic acid^1,2^** | **Amino acid intake recommended daily allowance for pregnancy^3^** |
| --- | --- | --- | --- |
| Histidine (mg) | 364.5 | 478.9 | 864 |
| Isoleucine (mg) | 834.9 | 716.4 | 1200 |
| Leucine (mg) | 1752.7 | 1564 | 2688 |
| Lysine (mg) | 1255.1 | 856.6 | 2448 |
| Methionine + cysteine (mg) | 673.6 | 565.3 | 1200 |
| Phenylalanine + tyrosine (mg) | 1313.4 | 1503.1 | 2112 |
| Threonine (mg) | 649.7 | 632.8 | 1248 |
| Tryptophan (mg) | 214.7 | 176.0 | 336 |
| Valine (mg) | 899.4 | 812.6 | 1488 |

Abbreviations: CVB, Centraal Veevoederbureau; RDA, recommended daily allowance; WFP, World Food Program

^1^ True ileal digestibility of indispensable amino acid content in each of the study foods based on values from CVB feed tables (CVB, Netherlands, http://vvdb.cvbdiervoeding.nl/Manage/Tools/VwCalc.aspx ).

^2^ Calculated based on a WFP supercereal product containing ~64% corn, ~24% soybean,~25g vegetable oil

^3^RDA based on mg/kg/day with an assumed weight of 48 kg. Reference values used Estimated Average Requirements (EAR) of healthy adults multiplied times 1.33 to account for increased protein demands of pregnancy. The RDA is based on 24% variability from the EAR. [1-3]

References:

1. Elango R, Ball RO. Protein and Amino Acid Requirements during Pregnancy. Adv Nutr. 2016;7(4):839S-44S. Epub 2016/07/17. doi: 10.3945/an.115.011817. PMID: 27422521

2. Kalhan SC. Protein metabolism in pregnancy. Am J Clin Nutr. 2000;71(5 Suppl):1249S-55S. Epub 2000/05/09. doi: 10.1093/ajcn/71.5.1249s. PMID: 10799398

3. Joint WHOFAOUNUEC. Protein and amino acid requirements in human nutrition. World Health Organ Tech Rep Ser. 2007;(935):1-265, back cover. Epub 2008/03/12. PMID: 18330140
