# Supplementary material for: A novel intervention combining supplementary food and infection control measures to improve birth outcomes in undernourished pregnant women in Sierra Leone: A randomized, controlled clinical effectiveness trial
Source: PLoS Med. 2021 Sep 28;18(9):e1003618. doi: 10.1371/journal.pmed.1003618 (PMC8478228; doi:10.1371/journal.pmed.1003618)
Supplement: S4 Table — (DOCX) [file pmed.1003618.s006.docx]

**S4 Table.** Maternal symptoms over first 4 weeks of enrollment, by treatment group^1^

|  | Intervention | | Standard | |  |  |
| --- | --- | --- | --- | --- | --- | --- |
| Symptom | n | Values | n | Values | P | Mean Difference (95% CI) |
| Nausea | 708 | 52(7.3) | 691 | 60(8.7) | 0.376 | 1.3(-1.7 to 4.3) |
| Fever^2^ | 706 | 139(19.7) | 691 | 142(20.5) | 0.689 | 0.9(-3.4 to 5.2) |
| Diarrhea^3^ | 706 | 31(4.4) | 690 | 35(5.1) | 0.614 | 0.7(-1.7 to 3.0) |
| Cough^4^ | 706 | 97(13.7) | 691 | 101(14.6) | 0.646 | 0.9(-2.9 to 4.6) |
| Rash^5^ | 705 | 39(5.5) | 690 | 26(3.8) | 0.128 | 1.7(-0.6 to 4.1) |
|  |  |  |  |  |  |  |

^1^Values expressed as absolute number (percentage).; *P* values calculated using Fischer exact test.

^2^Fever data missing, Intervention n=2

^3^Diarrhea data missing, Intervention n=2, Standard n=1

^4^Cough data missing, Intervention n=2

^5^Rash data missing, Intervention n=3, Standard n=1
