# Supplementary material for: A novel intervention combining supplementary food and infection control measures to improve birth outcomes in undernourished pregnant women in Sierra Leone: A randomized, controlled clinical effectiveness trial
Source: PLoS Med. 2021 Sep 28;18(9):e1003618. doi: 10.1371/journal.pmed.1003618 (PMC8478228; doi:10.1371/journal.pmed.1003618)
Supplement: S5 Table — (DOCX) [file pmed.1003618.s007.docx]

**S5 Table.** Adherence with Study Foods by Study Visit

|  | Intervention | Standard | | Difference (95% CI) | P |
| --- | --- | --- | --- | --- | --- |
| Visit 1 | 93.9(630) | 90.9 (592) | 3.0% (-0.02 to 6.0) | | 0.048 |
| Visit 2 | 95.3(607) | 92.9(560) | 2.4% (-0.3 to 5.3) | | 0.09 |
| Visit 3 | 96.9(558) | 93.2(522) | 3.7% (1.0 to 6.4) | | 0.006 |
| Visit 4 | 96.7(522) | 91.9(455) | 4.8% (1.8 to 7.9) | | 0.001 |
| Visit 5 | 97.3(472) | 94.8(417) | 2.5% (-0.1 to 5.4) | | 0.06 |
| Visit 6 | 96.8(422) | 96.9(375) | 0.1% (-2.5 to 2.9) | | 1.00 |
| Visit 7 | 97.9(369) | 96.4(320) | 1.5% (-1.2 to 4.5) | | 0.261 |
| Visit 8 | 98.4(302) | 97.9(279) | 0.5% (-2.2 to 3.3) | | 0.766 |
| Visit 9 | 98.3(225) | 97.7(210) | 0.6% (-2.7 to 4.1) | | 0.745 |

^1^Values expressed as mean *n* (%); *P* values calculated using Fisher’s Exact Test. The difference is given as the percentage-point difference between groups.
