# Supplementary material for: A novel intervention combining supplementary food and infection control measures to improve birth outcomes in undernourished pregnant women in Sierra Leone: A randomized, controlled clinical effectiveness trial
Source: PLoS Med. 2021 Sep 28;18(9):e1003618. doi: 10.1371/journal.pmed.1003618 (PMC8478228; doi:10.1371/journal.pmed.1003618)
Supplement: S6 Table — (DOCX) [file pmed.1003618.s008.docx]

**S6 Table.** Adherence with azithromycin and IPTp among pregnancies with singleton live births

|  | **Intervention** | **Standard** |
| --- | --- | --- |
|  | N=687 | N=657 |
| **Azithromycin** | n(%) | n(%) |
| No doses | 4 (0.6) |  |
| 1 dose | 210 (30.6) |  |
| 2 doses | 473 (68.9) |  |
| **IPTp** |  |  |
| No doses | 4 (0.6) | 8(1.2) |
| 1 dose | 16(2.3) | 25(3.8) |
| 2 doses | 49(7.1) | 73(11.1) |
| 3 doses | 119(17.3) | 548(83.4) |
| 4 doses | 160(23.3) | 3(0.5) |
| 5 doses | 177(25.8) |  |
| 6 doses | 133(19.4) |  |
| 7 doses | 29(4.2) |  |
|  |  |  |

Abbreviations: IPTp, intermittent preventative treatment for malaria in pregnancy
