# Supplementary material for: A novel intervention combining supplementary food and infection control measures to improve birth outcomes in undernourished pregnant women in Sierra Leone: A randomized, controlled clinical effectiveness trial
Source: PLoS Med. 2021 Sep 28;18(9):e1003618. doi: 10.1371/journal.pmed.1003618 (PMC8478228; doi:10.1371/journal.pmed.1003618)
Supplement: S7 Table — (DOCX) [file pmed.1003618.s009.docx]

**S7 Table.** Pregnancy outcomes, by treatment group^1^

|  | Intervention | | | Standard | |  |  |
| --- | --- | --- | --- | --- | --- | --- | --- |
| Outcome | *N* | Values | | *n* | Values | *P* | Difference (95% CI) |
| Live Birth | 751 | | 687(91.5) | 737 | 657(89.2) | 0.136 | 2.0(-1.1 to 5.0) |
| Twin Live Birth | 751 | | 8(1.2) | 737 | 6(0.8) | 0.593 | 0.3(-0.9 to 1.5) |
| Miscarriage | 751 | | 10(1.3) | 737 | 13(1.8) | 0.535 | 0.4(-1.0 to 1.9) |
| Stillbirth | 751 | | 15(2.0) | 737 | 18(2.5) | 0.601 | 0.4(-1.2 to 2.1) |
| Maternal Death^2^ | 751 | | 3(0.4) | 737 | 2(0.3) | >0.999 | 0.1(-0.7 to 1.0) |
| Lost to Follow-up | 751 | | 29(3.9) | 737 | 42(5.7) | 0.114 | 1.8(-0.5 to 4.1) |

^1^Values expressed as *n* (%); *P* values calculated using Fisher’s Exact Test. For outcomes reported as numbers and percentages of participants, the difference is given as the percentage-point difference between groups.

^2^Maternal death resulting in live born infants Intervention n=1, Standard n=1
