# Supplementary material for: A novel intervention combining supplementary food and infection control measures to improve birth outcomes in undernourished pregnant women in Sierra Leone: A randomized, controlled clinical effectiveness trial
Source: PLoS Med. 2021 Sep 28;18(9):e1003618. doi: 10.1371/journal.pmed.1003618 (PMC8478228; doi:10.1371/journal.pmed.1003618)
Supplement: S8 Table — (DOCX) [file pmed.1003618.s010.docx]

**S8 Table.** Linear mixed model results of maternal antenatal anthropometric outcomes^1^

|  | **Estimate** | **SE** | **t value** | **p** | **95% CI** |
| --- | --- | --- | --- | --- | --- |
| Maternal Weight Change, kg |  |  |  |  |  |
| Intercept | 5.39 | 1.10 | 4.903 | <0.001 | 3.24 to 7.55 |
| Time enrolled, wks | - 0.35 | 0.004 | 103.752 | <0.001 | -0.36 to -0.34 |
| Intervention | 2.21 | 1.54 | 1.435 | 0.152 | -0.81 to 5.22 |
| Time enrolled*Intervention | 0.03 | 0.01 | 6.193 | <0.001 | 0.02 to 0.04 |
| Maternal MUAC Change, cm |  |  |  |  |  |
| Intercept | 0.4 | 0.03 | 17.244 | <0.001 | 0.4 to 0.5 |
| Time enrolled, wks | 0.02 | 0.001 | 13.205 | <0.001 | 0.01 to 0.02 |
| Intervention | 0.1 | 0.04 | 3.861 | <0.001 | 0.1 to 0.2 |
| Time enrolled*Intervention | 0.01 | 0.002 | 6.113 | <0.001 | 0.01 to 0.01 |

Abbreviations: MUAC, mid-upper arm circumference; SE, standard error

^1^Linear midxed model constructed with fixed effects of the intervention, time enrolled in the study and their interaction were entered with individual participant variability accounted for as a random variable. p-values were estimated via t-tests using the Satterthwaite approximations to degrees of freedom.
