# Supplementary material for: A novel intervention combining supplementary food and infection control measures to improve birth outcomes in undernourished pregnant women in Sierra Leone: A randomized, controlled clinical effectiveness trial
Source: PLoS Med. 2021 Sep 28;18(9):e1003618. doi: 10.1371/journal.pmed.1003618 (PMC8478228; doi:10.1371/journal.pmed.1003618)
Supplement: S9 Table — (DOCX) [file pmed.1003618.s011.docx]

**S9 Table.** Linear mixed model results of maternal post-partum anthropometric outcomes^1^

|  | **Estimate** | **SE** | **T value** | **P** | **95% CI** |
| --- | --- | --- | --- | --- | --- |
| Maternal Weight, kg |  |  |  |  |  |
| Intercept | 46.08 | 0.1992 | 231.343 | <0.001 | 45.69 to 46.47 |
| Days since birth | -0.003 | 0.0007 | -4.188 | <0.001 | -0.005 to -0.002 |
| Intervention | 0.81 | 0.28 | 2.934 | 0.003 | 0.27 to 1.36 |
| Days since birth*Intervention | -0.0006 | 0.001 | -0.570 | 0.569 | -0.003 to 0.001 |
| Maternal MUAC, cm |  |  |  |  |  |
| Intercept | 22.8 | 0.1 | 433.257 | <0.001 | 22.7 to 22.9 |
| Days since birth | 0.003 | 0.0002 | 13.438 | <0.001 | 0.003 to 0.004 |
| Intervention | 0.1 | 0.1 | 1.313 | 0.189 | -0.04 to 0.2 |
| Days since birth*Intervention | 0.0001 | 0.0003 | 0.217 | 0.828 | -0.001 to 0.001 |

Abbreviations: MUAC, mid-upper arm circumference; SE, standard error

^1^Linear mixed model constructed with fixed effects of the intervention, time since birth and their interaction were entered with individual participant variability accounted for as a random variable. p-values were estimated via t-tests using the Satterthwaite approximations to degrees of freedom.
