# Supplementary material for: A novel intervention combining supplementary food and infection control measures to improve birth outcomes in undernourished pregnant women in Sierra Leone: A randomized, controlled clinical effectiveness trial
Source: PLoS Med. 2021 Sep 28;18(9):e1003618. doi: 10.1371/journal.pmed.1003618 (PMC8478228; doi:10.1371/journal.pmed.1003618)
Supplement: S10 Table — (DOCX) [file pmed.1003618.s012.docx]

**S10 Table**. Infant anthropometric outcomes by chronologic age

| Infant Length, cm by Chronological Age | | | |  | |  |  |
| --- | --- | --- | --- | --- | --- | --- | --- |
|  |  | **Intervention** | | **Standard** | |  |  |
|  | Chronological Age | n | Mean(SD) | n | Mean(SD) | p | Mean difference (95%CI) |
| **Birth Measurements**  Intervention=657  Standard=621 | < 7 days | 595 | 47.0±2.0 | 570 | 46.7±2.4 | **0.049** | 0.3(0.002 to 0.5) |
|  | 7-21 days | 62 | 49.5±2.3 | 51 | 48.5±2.8 | **0.035** | 1.0(-0.1 to 2.0) |
| **6-week measurements**  Intervention=625  Standard=578 | 4-8 weeks | 600 | 53.3±2.2 | 561 | 53.2±2.4 | 0.239 | 0.2(-0.1 to 0.4) |
|  | 8-9 weeks | 25 | 54.2±2.5 | 17 | 54.7±2.0 | 0.438 | -0.6(-2.1 to 0.9) |
| **3-month measurements**  Intervention=615  Standard=567 | 10-14 weeks | 530 | 58.0±2.3 | 483 | 57.7±2.4 | 0.143 | 0.2(-0.1 to 0.5) |
|  | 14-18 weeks | 85 | 59.5±2.5 | 84 | 59.2±2.3 | 0.52 | 0.2(-0.5 to 1.0) |
| **6-month measurements**  Intervention=588  Standard=539 | 20-28 weeks | 534 | 63.8±2.5 | 466 | 63.5±2.8 | 0.068 | 0.3(-0.02 to 0.6) |
|  | >28 weeks | 54 | 64.3±2.8 | 73 | 64.2±2.4 | 0.712 | 0.2(-o.7 to 1.1) |

Infant Weight, kg by Chronological Age

|  |  | **Intervention** | | **Standard** | |  |  |
| --- | --- | --- | --- | --- | --- | --- | --- |
|  | Chronological Age | n | Mean(SD) | n | Mean(SD) | p | Mean difference (95%CI) |
| **Birth Measurements**  Intervention=657  Standard=621 | < 7 days | 595 | 2.84±0.41 | 570 | 2.79±0.41 | **0.04** | 0.05(0.00 to 0.10) |
|  | 7-21 days | 62 | 3.24±0.50 | 51 | 3.02±0.64 | **0.046** | 0.22 (-0.00 to 0.43) |
| **6-week measurements**  Intervention=625  Standard=578 | 4-8 weeks | 600 | 4.29±0.64 | 561 | 4.21±0.67 | **0.037** | 0.08(0.01 to 0.16) |
|  | 8-9 weeks | 25 | 4.51±0.73 | 17 | 4.58±0.54 | 0.712 | -0.08(-0.50 to 0.34) |
| **3-month measurements**  Intervention=614  Standard=567 | 10-14 weeks | 529 | 5.42±0.77 | 483 | 5.36±0.78 | 0.210 | 0.06(-0.04 to 0.16) |
|  | 14-18 weeks | 85 | 5.77±0.75 | 84 | 5.67±0.80 | 0.401 | 0.10(-0.14 to 0.34) |
| **6-month measurements**  Intervention=586  Standard=539 | 20-28 weeks | 532 | 6.67±0.98 | 466 | 6.56±1.01 | 0.09 | 0.11(-0.02 to 0.23) |
|  | >28 weeks | 54 | 6.74±1.13 | 73 | 6.66±0.96 | 0.666 | 0.08(-0.29 to 0.45) |

Head Circumference, cm by Chronological Age

|  |  | **Intervention** | | **Standard** | |  |  |
| --- | --- | --- | --- | --- | --- | --- | --- |
|  | Chronological Age | n | Mean(SD) | n | Mean(SD) | p | Mean difference (95%CI) |
| **Birth Measurements**  Intervention=657  Standard=620 | < 7 days | 595 | 33.8±1.4 | 569 | 33.7±1.5 | **0.325** | 0.1(-0.1 to 0.3) |
|  | 7-21 days | 62 | 35.1±0.2 | 51 | 34.6±0.3 | 0.105 | 0.5(-0.1 to 1.2) |
| **6-week measurements**  Intervention=625  Standard=578 | 4-8 weeks | 600 | 37.2±1.3 | 561 | 37.1±1.4 | 0.303 | 0.1(-0.1 to 0.2) |
|  | 8-9 weeks | 25 | 37.5±1.4 | 17 | 37.9±1.0 | 0.311 | -0.4(-1.2 to 0.4) |
| **3-month measurements**  Intervention=615  Standard=567 | 10-14 weeks | 530 | 39.3±1.3 | 483 | 39.3±1.4 | 0.674 | 0.04(-0.1 to 0.2) |
|  | 14-18 weeks | 85 | 40.0±1.7 | 84 | 39.9±1.3 | 0.795 | 0.1(-0.4 to 0.5) |
| **6-month measurements**  Intervention=588  Standard=538 | 20-28 weeks | 534 | 41.9±1.4 | 466 | 41.8±1.5 | 0.248 | 0.1(-0.1 to 0.2) |
|  | >28 weeks | 54 | 42.1±1.6 | 72 | 42.2±1.4 | 0.603 | -0.1(-0.7 to 0.4) |

MUAC, cm by Chronological Age

|  |  | **Intervention** | | **Standard** | |  |  |
| --- | --- | --- | --- | --- | --- | --- | --- |
|  | Chronological Age | n | Mean(SD) | n | Mean(SD) | p | Mean difference (95%CI) |
| **Birth Measurements**  Intervention=657  Standard=621 | < 7 days | 595 | 9.8±0.7 | 570 | 9.7±0.8 | **0.031** | 0.1(0.01 to 0.2) |
|  | 7-21 days | 62 | 10.2±0.1 | 51 | 9.8±0.2 | 0.071 | 0.3(-0.03 to 0.7) |
| **6-week measurements**  Intervention=625  Standard=578 | 4-8 weeks | 600 | 11.7±1.1 | 561 | 11.6±1.1 | 0.191 | 0.1(-0.04 to 0.2) |
|  | 8-10 weeks | 25 | 11.8±0.9 | 17 | 11.9±1.0 | 0.681 | -0.1(-0.7 to 0.5) |
| **3-month measurements**  Intervention=615  Standard=567 | 10-14 weeks | 530 | 12.7±1.1 | 483 | 12.7±1.1 | 0.718 | 0.03(-0.1 to 0.2) |
|  | 14-18 weeks | 85 | 13.0±1.0 | 84 | 12.9±1.1 | 0.494 | 0.1(-0.2 to 0.4) |
| **6-month measurements**  Intervention=588  Standard=538 | 20-28 weeks | 534 | 13.4±1.2 | 466 | 13.3±1.3 | 0.548 | 0.1(-0.1 to 0.2) |
|  | >28 weeks | 54 | 13.5±1.3 | 72 | 13.2±1.1 | 0.21 | 0.3(-0.2 to 0.7) |

Abbreviations: MUAC, mid-upper arm circumference; SD, standard deviation; CI, confidence interval
