# Supplementary material for: A novel intervention combining supplementary food and infection control measures to improve birth outcomes in undernourished pregnant women in Sierra Leone: A randomized, controlled clinical effectiveness trial
Source: PLoS Med. 2021 Sep 28;18(9):e1003618. doi: 10.1371/journal.pmed.1003618 (PMC8478228; doi:10.1371/journal.pmed.1003618)
Supplement: S11 Table — (DOCX) [file pmed.1003618.s013.docx]

**S11 Table.** Linear mixed modelling of infant secondary outcomes to 6 months of life**^1^**

|  | **Estimate** | **SE** | **t value** | **p** | **95% CI** |
| --- | --- | --- | --- | --- | --- |
| Length, cm |  |  |  |  |  |
| Intercept | 47.7 | 0.1 | 449.458 | <0.001 | 47.5 to 47.9 |
| Age at measurement, days | 0.088 | 0.0004 | 206.670 | <0.001 | 0.087 to 0.089 |
| Male sex | 1.0 | 0.1 | 8.314 | <0.001 | 0.7 to 1.2 |
| Intervention | 0.3 | 0.1 | 2.533 | 0.0114 | 0.1 to 0.5 |
| Weight, kg |  |  |  |  |  |
| Intercept | 2.97 | 0.03 | 92.563 | <0.001 | 2.90 to 3.02 |
| Age at measurement | 0.02 | 0.0001 | 156.259 | <0.001 | 0.02 to 0.02 |
| Male sex | 0.30 | 0.03 | 8.960 | <0.001 | 0.23 to 0.36 |
| Intervention | 0.09 | 0.03 | 2.553 | 0.01 | 0.02 to 0.15 |
| MUAC, cm |  |  |  |  |  |
| Intercept | 10.2 | 0.04 | 234.922 | <0.001 | 10.1 to 10.3 |
| Age at measurement, days | 0.02 | 0.0002 | 85.994 | <0.001 | 0.02 to 0.02 |
| Male sex | 0.3 | 0.1 | 5.385 | <0.001 | 0.2 to 0.3 |
| Intervention | 0.1 | 0.1 | 2.058 | 0.0398 | 0.01 to 0.2 |
| Head Circumference, cm |  |  |  |  |  |
| Intercept | 34.2 | 0.1 | 552.371 | <0.001 | 34.1 to 34.3 |
| Age at measurement, days | 0.04 | 0.0003 | 171.060 | <0.001 | 0.04 to 0.04 |
| Male sex | 0.8 | 0.1 | 11.020 | <0.001 | 0.6 to 0.9 |
| Intervention | 0.1 | 0.1 | 1.589 | 0.112 | -0.03 to 0.2 |

Abbreviations: MUAC, mid-upper arm circumference; SE, standard error

^1^ Linear mixed model was constructed with fixed effects of the intervention, age at measurement, and infant sex, and their interaction with individual participant variability accounted for as a random variable. p-values were estimated via t-tests using the Satterthwaite approximations to degrees of freedom.
