# Supplementary material for: A novel intervention combining supplementary food and infection control measures to improve birth outcomes in undernourished pregnant women in Sierra Leone: A randomized, controlled clinical effectiveness trial
Source: PLoS Med. 2021 Sep 28;18(9):e1003618. doi: 10.1371/journal.pmed.1003618 (PMC8478228; doi:10.1371/journal.pmed.1003618)
Supplement: S12 Table — (DOCX) [file pmed.1003618.s014.docx]

**S12 Table.** Infant mortality among singleton infants, by treatment group^1^

|  | Intervention  *n*= 687 | Standard  *n*= 657 |  |  |
| --- | --- | --- | --- | --- |
| Outcome | Values^1^ | Values^1^ | *p* | Difference (95%CI) |
| Infant Death, total | 35(5.6) | 53(8.9) | **0.027** | 3.3%(0.2 to 6.4) |
| Death within 28 days of delivery^2^ | 13(1.9) | 28(4.3) | **0.016** | 2.4%(0.3 to 4.4) |
| Death occurred > 28 days after delivery^3^ | 22(3.5) | 25(4.2) | 0.555 | 0.7%(-1.7% to 3.0%) |
| Lost to Follow-up | 64(9.3) | 65(9.9) | 0.781 | 0.6%(-2.7 to 3.8) |

^1^Values expressed as *n* (%); *P* values calculated using Fisher’s Exact Test.

^2^Lost to follow-up after birth measure Intervention n=6, Standard n=5. All other infants with 6-week survival data.

^3^Includes only infants with 6-month survival follow-up data. Infants lost to follow-up excluded.

All infants included in Kaplan-Meier survival analysis and censored when appropriate.
