# Supplementary material for: A novel intervention combining supplementary food and infection control measures to improve birth outcomes in undernourished pregnant women in Sierra Leone: A randomized, controlled clinical effectiveness trial
Source: PLoS Med. 2021 Sep 28;18(9):e1003618. doi: 10.1371/journal.pmed.1003618 (PMC8478228; doi:10.1371/journal.pmed.1003618)
Supplement: S13 Table — (DOCX) [file pmed.1003618.s015.docx]

**S13 Table**. Maternal and pregnancy outcomes by vaginal dysbiosis status, among the intervention group^1^

|  | | Vaginal dysbiosis Positive | | | | Vaginal dysbiosis Negative | | |  | |  | | |
| --- | --- | --- | --- | --- | --- | --- | --- | --- | --- | --- | --- | --- | --- |
| Outcome | | n | Mean | | | n | Mean | | | P | | Mean Difference(95% CI) | |
| **Maternal Outcomes** | | | | | | | | | | | | | |
| Weight gain, kg | | 415 | 0.4±0.8 | | | 267 | 0.6±0.9 | | | 0.055 | | -0.1(-0.3 to 0.003) | |
| MUAC gain, cm | | 414 | 5.08±3.50 | | | 267 | 5.86±4.08 | | | 0.007 | | -0.79(-1.36 to -0.21) | |
| Fundal Height, cm | |  |  | | |  |  | | |  | |  | |
| **Pregnancy Outcomes^2,3^** | | | | | | | | | | | | | |
| Live Birth | 420 | | | 404(96.2) | 280 | | | 270(96.6) | 0.842 | | | | 0.4%(-2.6 to 3.7) |
| Twin Live Birth | 420 | | | 3(0.7) | 280 | | | 5(1.8) | 0.283 | | | | 1.0%(-1.6 to 3.0) |
| Miscarriage | 420 | | | 1(0.2) | 280 | | | 2(0.7) | 0.567 | | | | 0.5%(-1.7 to 1.9) |
| Still Birth | 420 | | | 11(2.6) | 280 | | | 3(1.1) | 0.178 | | | | 1.5%(-1.1 to 3.8) |
| Maternal Death^4^ | 420 | | | 3(0.7) | 280 | | | 0(0) | 0.279 | | | | 0.7%(-1.1 to 2.3) |
| Infant Deaths | 404 | | | 14(3.5) | 270 | | | 10(3.6) | >0.999 | | | | 0.1%(-2.9 to 3.5) |

Abbreviations: CI, confidence interval; MUAC, mid-upper arm circumference; SD, standard deviation

^1^Values expressed as mean ± SD; *P* values calculated using independent t-test.

^2^Values expressed as *n* (%); *P* values calculated using Fisher’s Exact Test.

^3^23 Live births with missing, incomplete or delivery prior to BV status data collection.

^4^2 maternal deaths after delivery.
