# Supplementary material for: A novel intervention combining supplementary food and infection control measures to improve birth outcomes in undernourished pregnant women in Sierra Leone: A randomized, controlled clinical effectiveness trial
Source: PLoS Med. 2021 Sep 28;18(9):e1003618. doi: 10.1371/journal.pmed.1003618 (PMC8478228; doi:10.1371/journal.pmed.1003618)
Supplement: S14 Table — (DOCX) [file pmed.1003618.s016.docx]

**S14 Table.** Infant birth outcomes by maternal vaginal dysbiosis status, among the intervention group^1^

| \|  \| Vaginal dysbiosis  Positive \| \| Vaginal dysbiosis Negative \| \|  \|  \| \| \| --- \| --- \| --- \| --- \| --- \| --- \| --- \| --- \| \| Outcome \| n \| Mean \| n \| Mean \| P \| \| Mean Difference(95% CI) \| \| | | | | | | |
| --- | --- | --- | --- | --- | --- | --- | --- | --- | --- | --- | --- | --- | --- | --- | --- | --- | --- | --- | --- | --- | --- | --- | --- |
| Weight, kg | 404 | 2.90±0.45 | 260 | 2.87±0.47 | 0.475 | 0.03(-0.05 to 0.10) |
| Length, cm | 404 | 47.2±2.4 | 260 | 47.4±2.1 | 0.173 | -0.2(-0.6 to 0.1) |
| MUAC, cm | 404 | 9.9±0.7 | 260 | 9.8±0.9 | 0.22 | 0.1(-0.05 to 0.2) |
| Head circumference, cm | 404 | 34.1±1.4 | 260 | 33.8±1.5 | 0.016 | 0.3(0.1 to 0.5) |
| Weight-for-length,z-score | 340 | 0.13±1.16 | 233 | -0.07±1.06 | 0.038 | 0.20(0.01 to 0.39) |
| Length-for-age, z-score | 403 | -1.51±1.11 | 260 | -1.40±1.01 | 0.196 | -0.11(-0.28 to 0.06) |
| Weight-for-age, z-score | 403 | -0.98±0.99 | 260 | -1.06±0.96 | 0.3 | 0.08(-0.07 to 0.23) |

Abbreviations: CI, confidence interval; MUAC, mid-upper arm circumference; SD, standard deviation

^1^Values expressed as mean ± SD; *P* values calculated using independent t-test
