# Supplementary material for: A novel intervention combining supplementary food and infection control measures to improve birth outcomes in undernourished pregnant women in Sierra Leone: A randomized, controlled clinical effectiveness trial
Source: PLoS Med. 2021 Sep 28;18(9):e1003618. doi: 10.1371/journal.pmed.1003618 (PMC8478228; doi:10.1371/journal.pmed.1003618)
Supplement: S15 Table — (DOCX) [file pmed.1003618.s017.docx]

**S15 Table**. Trials giving azithromycin in pregnancy with > 1200 participants in low- and middle-income settings

| **Trial** | **Location** | **Azithromycin timing** | **Gestational weight gain** | **Premature delivery** | **Malaria** | **Birth weight** | **Low birth weight** | **Birth length** | **Mortality** | **Sexual transmitted diseases** |
| --- | --- | --- | --- | --- | --- | --- | --- | --- | --- | --- |
| Goldenberg[1] | Zambia, Malawi, Tanzania  2001-03 | Once 24 wk | NR | Fundal ht ND | ND | MD 5 g NS | ND | NR | ND | Trichomoniasis (only HIV infected) ↓ and BV (HIV infected and uninfected) ↓ |
| Gray[2] | Uganda | Once, whenever pregnancy identified equally divided | NR | RR=0.77 NS | NR | NR | Estimated by CC and HC  RR = 0.76 | NR | < 7d RR=0.83 | Trichomoniasis, chlamydia, vaginal dysbiosis  all ↓ |
| Van den Broek[3] | Malawi, 2004-05 | Twice,  16-24 wk and 28-32 wk | NR | OR 0.96 NS | OR 1.11  ND | MD 40 g NS | NR | NR | Perinatal OR=0.85 NS | NR |
| Luntamo[4] | Malawi  2003-06 | Twice, enroll and 28-34 wk | NR | RR=0.66  ARR= 6.1% | Immediate effect seen not sustained | MD 140 g | RR = 0.61 ARR= 5.1% | MD  0.4 -0.7 cm sustained throughout follow-up (1-60 mo) | Perinatal RR=0.84 NS | Trichomoniasis ↓  ND chlamydia or Gonorrhea |
| Unger[5] | Papua New Guinea 2009-13 | 1-3 doses separated by 1 mo | 419 g/wk | RR=0.57 | RR =0.57  AAR= 1.7% | MD 42 g | RR =0.74  ARR= 4.5% | ND at postnatal visit | Neonatal RR=0.58 NS | Gonorrhea ↓ |
| Hendrixson | Sierra Leone 2017-20 | Twice, 2^nd^ and 3^rd^ trimesters | 379 g/wk | Fundal ht  < 37 cm reduced | ND | MD 70 g | Diff 1.3%  RR=0.88  NS | MD 0.3 cm | Neonatal  HR=0.46  Infant  HR=0.62 | BV poorly controlled |

Abbreviations: Wk: week; NR: not reported; ND: no difference, NS: not significant; RR: relative risk; HR: hazard ratio; CC: chest circumference; HC: head circumference OR: Odd Ratio; MD: mean difference; ARR: absolute risk reduction; ↓: reduced

References:

1. Goldenberg RL, Mwatha A, Read JS, Adeniyi-Jones S, Sinkala M, Msmanga G, et al. The HPTN 024 Study: the efficacy of antibiotics to prevent chorioamnionitis and preterm birth. Am J Obstet Gynecol. 2006;194(3):650-61. Epub 2006/03/09. doi: 10.1016/j.ajog.2006.01.004. PMID: 16522393

2. Gray RH, Wabwire-Mangen F, Kigozi G, Sewankambo NK, Serwadda D, Moulton LH, et al. Randomized trial of presumptive sexually transmitted disease therapy during pregnancy in Rakai, Uganda. Am J Obstet Gynecol. 2001;185(5):1209-17. Epub 2001/11/22. doi: 10.1067/mob.2001.118158. PMID: 11717659

3. van den Broek NR, White SA, Goodall M, Ntonya C, Kayira E, Kafulafula G, et al. The APPLe study: a randomized, community-based, placebo-controlled trial of azithromycin for the prevention of preterm birth, with meta-analysis. PLoS Med. 2009;6(12):e1000191. Epub 2009/12/04. doi: 10.1371/journal.pmed.1000191. PMID: 19956761

4. Luntamo M, Kulmala T, Cheung YB, Maleta K, Ashorn P. The effect of antenatal monthly sulphadoxine–pyrimethamine, alone or with azithromycin, on foetal and neonatal growth faltering in Malawi: a randomised controlled trial. Tropical Medicine & International Health. 2013;18(4):386-97.

5. Unger HW, Ome-Kaius M, Wangnapi RA, Umbers AJ, Hanieh S, Suen CS, et al. Sulphadoxine-pyrimethamine plus azithromycin for the prevention of low birthweight in Papua New Guinea: a randomised controlled trial. BMC Med. 2015;13:9. Epub 2015/01/17. doi: 10.1186/s12916-014-0258-3. PMID: 25591391
